# Supplementary figures and images for: Fecal Microbiota Transplantation Using Donor Stool Obtained from Exercised Mice Suppresses Colonic Tumor Development Induced by Azoxymethane in High-Fat Diet-Induced Obese Mice
Source: Microorganisms. 2025 Apr 27;13(5):1009. doi: 10.3390/microorganisms13051009 (PMC12114393; doi:10.3390/microorganisms13051009)

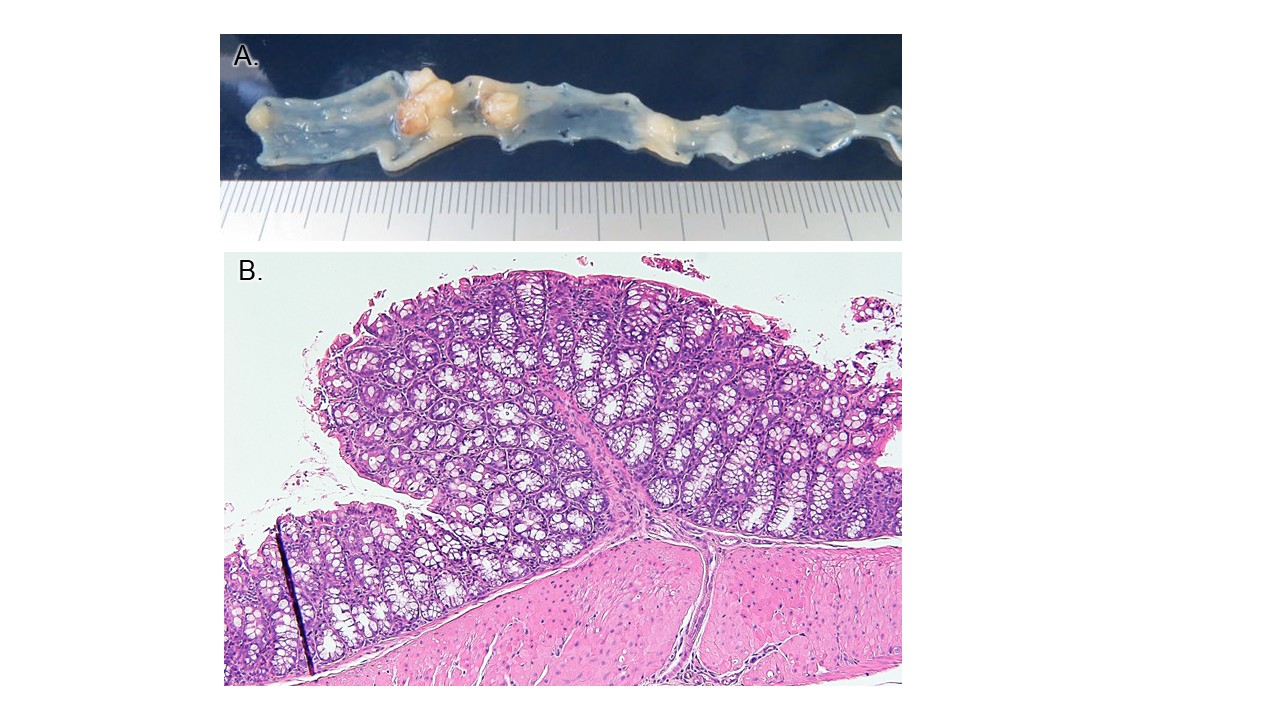

Supplement: Supplementary file 1 [file microorganisms-13-01009-s001.zip › microorganisms-3521377-supplementary.jpg]
